# Reproducibility of coronary artery calcium quantification on dual-source CT and dual-source photon-counting CT: a dynamic phantom study

**DOI:** 10.1007/s10554-022-02540-z

**Published:** 2022-02-03

**Authors:** Niels R. van der Werf, Ronald Booij, Marcel J. W. Greuter, Daniel Bos, A. van der Lugt, R. P. J. Budde, Marcel van Straten

**Affiliations:** 1 Department of Radiology & Nuclear Medicine, Erasmus University Medical Center, Rotterdam, The Netherlands; 2 Department of Radiology, University of Groningen, University Medical Center Groningen, Groningen, The Netherlands; 3 Department of Robotics and Mechatronics, University of Twente, Enschede, The Netherlands

**Keywords:** X-ray computed tomography, Calcium, Coronary vessels, Imaging phantoms

## Abstract

To systematically compare coronary artery calcium (CAC) quantification between conventional computed tomography (CT) and photon-counting CT (PCCT) at different virtual monoenergetic (monoE) levels for different heart rates. A dynamic (heart rates of 0, < 60, 60–75, and > 75 bpm) anthropomorphic phantom with three calcification densities was scanned using routine clinical CAC protocols with CT and PCCT. In addition to the standard clinical protocol of 70 keV, PCCT images were reconstructed at monoE levels of 72, 74, and 76 keV. CAC was quantified using Agatston, volume, and mass scores. Agatston scores 95% confidence intervals (CI) were calculated and compared between PCCT and CT. Volume and mass scores were compared with physical quantities. For all CAC densities, routine clinical protocol Agatston scores of static CAC were higher for PCCT compared to CT. At < 60 bpm, Agatston scores at 74 and 76 keV reconstructions were reproducible (overlapping CI) for PCCT and CT. Increased heart rates yielded different Agatston scores for PCCT in comparison with CT, for all monoE levels. Low density CAC volume scores showed the largest deviation from physical volume, with mean deviations of 59% and 77% for CT and PCCT, respectively. Overall, mass scores underestimated physical mass by 10%, 38%, and 59% for low, medium, and high density CAC, respectively. PCCT allows for reproducible Agatston scores for dynamic CAC (< 60 bpm) when reconstructed at monoE levels of 74 or 76 keV, regardless of CAC density. Deviations from physical volume and mass were, in general, large for both CT and PCCT.

## Introduction

Quantification of coronary artery calcium (CAC) on computed tomography (CT) is strongly related to future adverse cardiovascular events [1, 2]. Consequently, the Agatston score methodology is clinically used for cardiovascular risk stratification [2–5]. Monitoring of CAC over time has also become clinically important as its progression is associated with an increased risk cardiovascular events [6]. To be able to define CAC progression, therefore, reproducible CAC scores are essential.

Currently, a new CT technology is finding its way to the clinic: photon-counting CT (PCCT) [7–14]. In contrast to conventional CT, PCCT detectors allow for photon energy discriminating measurements. In turn, this enables counting the number of photons within predefined energy bins. In comparison with conventional CT, PCCT also exhibit superior inherent spatial resolution due to smaller detector elements. This is possible thanks to the lack of reflecting layers which are required to minimize crosstalk of visible light between neighboring elements, as for PCCT photons are directly converted to electronic pulses [12]. Also, because of high photon flux for CT, PCCT detector elements are small to avoid problems caused by pulse pile-up effects [15–17].

Previous studies assessed the potential of CAC assessment with different PCCT systems and found good Agatston score reproducibility and improved volume estimation for PCCT [13, 18–22]. However, these studies did not systematically assess the influence of coronary artery movement during the scan phase for different heart rates. This movement is often erroneously assumed to be nonexistent thanks to reconstructing in a specific phase of the cardiac cycle. However, the impact of coronary artery motion on resulting Agatston scores can be large [23–25].

For a recently clinically released whole body PCCT, the clinical CAC protocol employs virtual monoenergetic (monoE) reconstructions at 70 keV for acquisitions at a tube potential of 120 kVp. It is, however, unknown if this monoE level on PCCT results in reproducible CAC scores obtained previously with conventional CT for dynamic CAC. The aim of the current study was, therefore, to systematically assess CAC quantification reproducibility between conventional CT and PCCT at different monoE levels for different heart rates.

## Methods

### Phantom

To assess CAC quantification, an anthropomorphic thorax phantom (QRM Thorax, PTW) was scanned on a conventional CT (SOMATOM Force, Siemens Healthineers) and a PCCT (NAEOTOM Alpha, Siemens Healthineers) system. The dimensions of the thorax phantom were increased with a fat tissue equivalent large extension ring (QRM Extension Ring, PTW), to resemble a large sized patient [26]. Two artificial cylindrical coronary arteries were translated in a water compartment, which was positioned at the center of the thorax phantom. The artificial arteries contained calcifications with equal dimensions (10 mm length, 5 mm diameter) and different hydroxyapatite (HA) densities: 196 ± 3, 380 ± 2 and 800 ± 2 mg/cm^3^, indicated as low, medium, and high density, respectively. The artificial coronary arteries were translated in the x–y plane at constant velocities of 0, 10, 20, and 30 mm/s. These velocities corresponded to grouped heart rates of 0, < 60, 60–75, and > 75 beats per minute (bpm), as defined by Husmann et al. [23–25]. The movement of the coronary arteries was performed with a motion simulator (QRM Sim2D, PTW). The electrocardiogram output of the motion simulator was used to synchronize linear phantom movement and CT data acquisition. Each scan setup was repeated five times, with small manual translations of the phantom between each scan.

### Data acquisition and reconstruction

On both CT systems, routine clinical CAC scoring protocols were used (Table 1). Additional to the clinical 70 keV reconstruction, PCCT data was reconstructed at monoE levels of 72, 74, and 76 keV.

**Table 1:** Acquisition and reconstruction parameters of clinical protocols for CT and PCCT

Parameter	CT	PCCT
CT system	Force	Alpha
Technique	Axial	Axial
Tube voltage [kVp]	120	120
Collimation [mm]	96 × 0.6 (with flying focal spot)	144 × 0.4
Rotation time [s]	0.25	0.25
Field of view [mm]	220	220
Slice thickness/increment [mm]	3.0/1.5	3.0/1.5
Reconstruction kernel	Qr36	Qr36
Matrix size [pixels]	512 × 512	512 × 512
Reconstruction	FBP	FBP^a^
Virtual monoenergetic level [keV]	–	70
Repetitions	5	5
Scan length	10 cm	10 cm
Volumetric CT dose index [mGy]	3.93^b^	4.06^c^

^a^*FBP* filtered back projection. The setting used was actually Quantum Iterative Reconstruction (QIR, Siemens Healthineers) off, which is comparable to a conventional reconstruction in terms of the expected noise level

^b^With static tube current time product based on reference tube current time product with dose optimization slider on position 5 (default calcium scoring protocol) (CARE Dose4D, Siemens Healthineers)

^c^With static tube current time product based on the vendor recommended reference tube current modulation (CARE keV, Siemens Healthineers) IQ level 16

### Data analysis

CAC was expressed as Agatston, volume, and mass scores on all reconstructions using the vendor specific CAC scoring parameters which were implemented in a previously validated in-house developed Python script [27]. These parameters included the use of a calcium scoring threshold of 130 Hounsfield Units (HU) to discriminate calcium-containing voxels from background material. No minimum number of connected voxels was used to include voxels of a specific calcification in the Agatston score. For volume score calculations, three dimensional isotropic voxels were used. Finally, a CT-specific calibration factor was used to calculate the mass score, as described previously [26].

For each heart rate and CAC density, PCCT Agatston scores were compared to the reference (Agatston scores from conventional CT) to assess CAC quantification reproducibility. Confidence intervals (95%) were used to assess differences, with overlapping CI deemed as comparable. Mean and 95% confidence interval volume and mass scores were compared with the vendor specified physical volume (196.3 mm^3^) or mass (38.5, 74.6, or 157.1 mg for low, medium, or high-density CAC respectively) of the phantom.

## Results

**Fig. 1:**
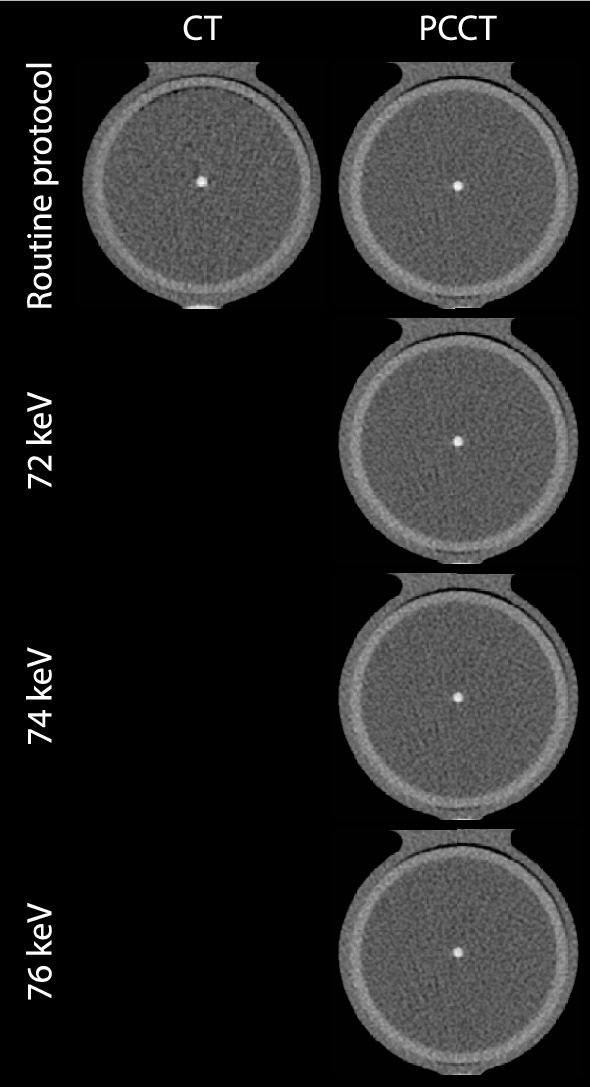
Representative images for the medium density CAC for both CT (left) and PCCT (right) at 60–75 bpm. The upper row represents the routine clinical protocol, with a monoE level of 70 keV for PCCT. Increased monoE levels images are shown in the lower rows

For CT and PCCT, representative images for both the routine clinical protocol and increased monoE levels for the medium density CAC at 60–75 bpm are shown in Fig. 1.

### Agatston scores

**Fig. 2:**
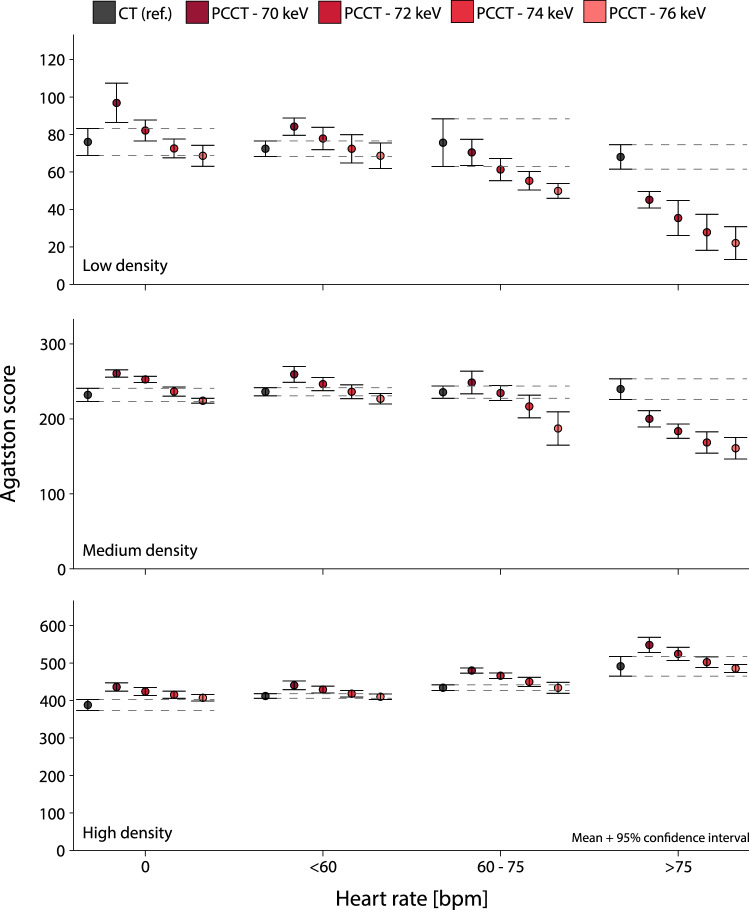
Mean and 95% confidence interval Agatston scores for low (top), medium (middle), and high (bottom) CAC density, for both conventional CT and PCCT at four monoE levels. For each density, data is presented for 0, < 60, 60–75 and > 75 beats-per-minute. For each heart rate, dashed lines indicate the 95% confidence intervals of the reference

For all CAC densities, static Agatston scores on PCCT were higher for the routine clinical protocol (i.e. 70 keV) in comparison with CT (Fig. 2). PCCT Agatston scores decreased with increasing monoE levels. In contrast to CT, low density CAC Agatston scores decreased for PCCT at increased heart rate. For high density CAC, PCCT scores were higher than CT Agatston scores at increased heart rate.

At 0 bpm, low density CAC Agatston scores for PCCT were comparable to CT for 72, 74 and 76 keV. For medium density CAC, monoE levels of 74 and 76 keV resulted in comparable Agatston scores between both CT systems. For high density CAC, only 76 keV resulted in comparable scores.

At < 60 bpm, 74 and 76 keV reconstructions resulted in comparable Agatston scores for PCCT in comparison with CT. For low and medium density CAC, a monoE level of 72 keV did also result in similar Agatston scores.

At 60–75 bpm, none of the monoE levels resulted in comparable Agatston scores to the reference for all CAC densities simultaneously. Comparable Agatston scores were found for low density CAC when reconstructed with 70 or 72 keV. Medium density CAC Agatston scores were comparable for 70, 72, and 74 keV reconstructions, while high density CAC Agatston scores were comparable 74 and 76 keV.

For > 75 bpm, low and medium density CAC Agatston scores were lower than the reference scores for all monoE levels. For high density CAC, all increased monoE levels (72–76 keV) resulted in similar Agatston scores in comparison with the reference.

### Volume and mass scores

**Fig. 3:**
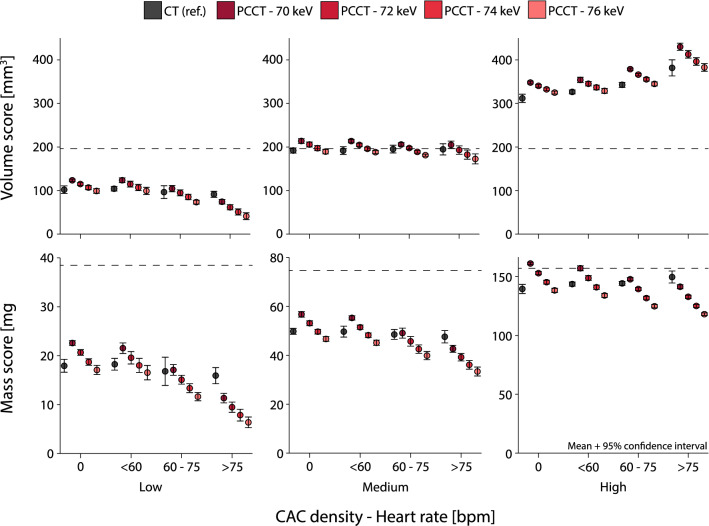
Mean and 95% confidence interval volume (top) and mass scores (bottom) for low (left), medium (middle), and high (right) CAC density, for both conventional CT and PCCT at four monoE levels. For each density, data is presented for 0, < 60, 60–75 and > 75 beats-per-minute. Physical phantom quantities (volume of 196.3 mm^3^ and mass of 38.5, 74.6, or 157.1 mg for low, medium, or high density CAC respectively) are indicated with dashed lines

Low and high density CAC volume scores showed large deviations from physical volume for all heart rates and reconstructions (Fig. 3). For CT with the clinical protocol, static mean volume scores deviated from physical volume by − 48%, − 2%, and 59% for low, medium, and high density CAC, respectively. For PCCT, the clinical protocol resulted in mean deviations from physical volume by − 37%, 9%, and 77%. Volume scores decreased with increasing monoE levels. For low density CAC, deviations from physical volume increased, while high density CAC deviations from physical volume decreased. All volume scores from low and high density CAC were different from the physical volume, irrespective of CT system, heart rate, and monoE level. For medium density CAC, CT volume scores were comparable with the physical volume, regardless of heart rate. For PCCT, 74 keV resulted in similar volume approximation for 0 and < 60 bpm, while 72 keV was needed for increased heart rates.

For CT, mass scores underestimated physical mass regardless of CAC density or heart rate (Fig. 3). Mean deviations from physical mass for static CAC were − 53%, − 33%, and − 11% for low, medium, and high density CAC. For PCCT, physical mass was underestimated by low and medium density CAC mass scores, regardless of heart rate or monoE level. For high density CAC, mass scores were comparable to the reference only for < 60 bpm, reconstructed at 70 keV. All other heart rates and monoE levels resulted in differences in Mass score with the reference.

## Discussion

This study demonstrated that for our dynamic phantom setup current clinical PCCT protocols for calcium scoring result in different Agatston scores for all heart rates compared to CT. However, when reconstructed at an increased monoE level of 74 or 76 keV, PCCT Agatston scores for heart rates of < 60 bpm were comparable with the reference conventional CT, regardless of CAC density. At higher heart rates a larger deviation in Agatston scores was seen. For both CT and PCCT, deviations from physical volume were large for low and high density CAC, while deviations from physical mass were large for low and medium density CAC.

This study is, to the best of our knowledge, the first to systematically assess Agatston score reproducibility as a function of monoE levels for dynamic calcifications. For our dual-source PCCT system, monoE reconstructions are available at high temporal resolution. This is in contrast to previous dual-source solutions, where temporal resolution had to be sacrificed to obtain spectral data.

Previous studies did also assess the potential of PCCT for static CAC assessment. A recent study by Eberhard et al. found accurate CAC scoring for monoE reconstructions for both phantom and patient data [20]. Their study, however, did not use a dynamic phantom or intermediate monoE levels, as only 60–75 keV in steps of 5 keV were used. Furthermore, only summed Agatston scores for three CAC densities were evaluated. Symons et al. reported accurate CAC score assessment for PCCT in comparison with conventional CT for ex-vivo hearts [19]. These CAC were, however, static and with an unknown density. For a different PCCT vendor, clinical CAC protocols (with non-monoE reconstructions) were shown to result in comparable Agatston scores for PCCT in comparison with conventional CT [13]. The paper, however, also described improve CAC detection for PCCT in comparison with CT, potentially due to increased spatial resolution and associated reduced partial volume effects. In turn, for PCCT, this may result in differences in Agatston score with conventional CT, hampering the assessment of CAC burden progression. For a previous (prototype) version of our PCCT system, Sandstedt et al. found increased volume quantification with PCCT for static CAC in comparison with conventional CT due to reduced calcium blooming artefacts [18]. In our study, only for low density CAC, volume quantification was superior for PCCT. However, our data was based on clinical CAC protocols, while high resolution image data was used by Sandstedt et al. [18].

This study has limitations. First, the results were based on a single sized anthropomorphic phantom with artificial coronary arteries. Although this phantom size resembled a large sized patient, it is well known that patient or phantom size affects beam hardening and consequently CT numbers [26]. Current results may, therefore, not be transferable to smaller patient sizes. The used CAC densities were mixtures of pure HA and so-called solid water and were in the range observed in patients [28]. Second, only linear motion in one direction perpendicular to the scan-plane was used to simulate the complex 3D in-vivo motion of coronary arteries [23]. This linear motion was deemed sufficient, due to the short scan time and fast rotation times of the CT gantry. Third, the current study assessed reproducibility of PCCT Agatston scores in comparison with conventional CT Agatston scores with overlapping CI. As the inherent reproducibility of Agatston scores is suboptimal, the precision of these scores may be low. In turn, Agatston scores with large CI may be classified as comparable between both CT systems, while absolute differences are large. The current setup was deemed appropriate for the focus of the current study was, namely to assess potential differences in Agatston scores as a function of a change in CT system. Fourth, only one PCCT system was used for the current study. Currently, however, this is the only clinically available system which can provide high temporal resolution monoE reconstructions.

PCCT allows for reproducible Agatston scores in comparison with conventional CT for dynamic CAC with a heart rate of < 60 bpm when reconstructed at a monoE level of 74 or 76 keV, regardless of CAC density. Deviations from physical volume and mass were, in general, large for both CT systems. In general, deviations from physical volume and mass were large.
